# Relative corneal refractive power shift and inter-eye differential axial growth in children with myopic anisometropia treated with bilateral orthokeratology

**DOI:** 10.1007/s00417-023-06301-z

**Published:** 2023-11-06

**Authors:** Weiping Lin, Na Li, Jiahe Liu, Bin Zhang, Ruihua Wei

**Affiliations:** 1 Tianjin Key Laboratory of Retinal Functions and Diseases, Tianjin Branch of National Clinical Research Center for Ocular Disease, Eye Institute and School of Optometry, Tianjin Medical University Eye Hospital, Tianjin, China; 2 College of Optometry, Nova Southeastern University, Davie, FL USA

**Keywords:** Myopia, Anisometropia, Orthokeratology, Relative corneal refractive power shift, Axial growth

## Abstract

**Purpose:**

To investigate the relationship between relative corneal refractive power shift (RCRPS) and axial length growth (ALG) in bilateral myopic anisometropes treated with orthokeratology.

**Methods:**

A total of 102 children with myopic anisometropia in this prospective interventional study were randomly assigned to the spectacle group and orthokeratology group. Axial length (AL) and corneal topography was measured at baseline and the 12-month follow-up visit. ALG was defined as the difference between the two measurements, and RCRPS profiles were calculated from two axial maps obtained.

**Results:**

In the orthokeratology group, the ALG in the more myopic eye (0.06 ± 0.15 mm) was significantly smaller than that in the less myopic eye (0.15 ± 0.15 mm, *p* < 0.001), and the interocular difference in AL significantly decreased following 1-year treatment, from 0.47 ± 0.32 to 0.38 ± 0.28 mm (*p* < 0.001). However, in the spectacle group, the ALG was similar between the two eyes, and the interocular difference in AL did not change significantly over one year (all *p* > 0.05). The interocular difference in ALG in the orthokeratology group was significantly correlated with the interocular difference in RCRPS (dRCRPS, *β*=−0.003, *p* < 0.001) and the interocular difference in baseline AL (*β*=−0.1179, *p* < 0.001), with *R*^2^ being 0.6197.

**Conclusion:**

Orthokeratology was effective in decreasing the magnitude of anisometropia. The interocular variation in RCRPS is an important factor accounting for the reduction of interocular ALG difference in anisomyopic children post-orthokeratology. These results provide insight into establishing eye-specific myopia control guidelines during orthokeratology treatment for myopic anisometropes.
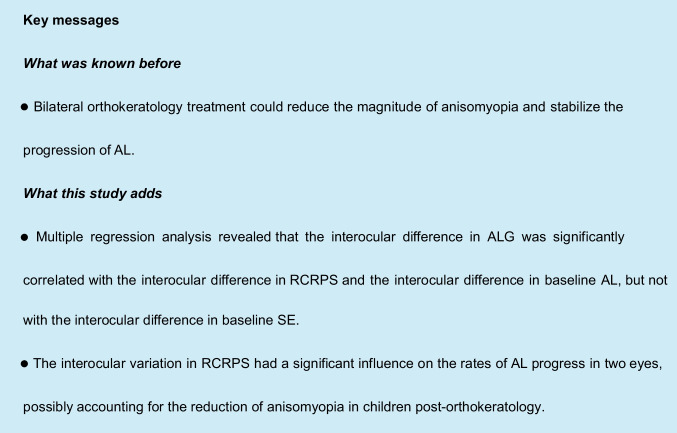

## Introduction

Orthokeratology lenses are rigid contact lenses with reverse geometry on their back surface. Overnight wear of these lenses results in a flattening of the central portion and steepening of the peripheral portion of the cornea [1–3]. The flattened central portion of the cornea improves daytime vision, while the steepened peripheral portion induces a relative corneal refractive power shift (RCRPS) from the baseline, leading to myopic defocus on the peripheral retina. This has been suggested as the underlying mechanism of the retardation of axial growth by orthokeratology [4–6]. In comparison with single vision spectacles, orthokeratology lenses can retard axial growth by as much as 32–63% in patients [7–12]. Although orthokeratology lenses are widely used in clinical practice, the effectiveness of these lenses for myopic control varies greatly in different conditions.

Anisometropia refers to a condition of an inter-eye difference in refractive status, mainly due to an imbalance in axial growth between the two eyes of an individual [13]. Factors, such as myopia, scoliosis screening positive, hyperopia, female sex, older age, higher weight, and unscientific near work habits, have been referred to as risk factors for anisometropia [14, 15]. Typical myopic anisometropia is defined as an interocular difference in spherical equivalent (SE) of one diopter or more, affecting approximately 10% of adolescents [16, 17]. Effective management of anisometropia involves balancing visual acuity between the two eyes, slowing myopia progression, and reducing the magnitude of anisometropia [18]. Spectacles are the most common optical correction but their full-correction prescription may be less tolerated in clinic when the magnitude of anisometropia is large. Single-vision spectacles have been proved to show no effect on myopia control and anisometropia reduction [19, 20]. Orthokeratology has been reported as an effective tool for managing myopic anisometropia. It has been shown to correct interocular differences in refractive errors and slow axial growth in both eyes compared to wearing single-vision spectacles [20–27]. However, no prior study has analyzed the possible influencing factors accounting for different axial length growth (ALG) in two eyes of anisometropic children following orthokeratology treatment.

Peripheral retinal myopic defocus has been shown to play an important role in slowing down the myopic progress [4, 28]. As detecting peripheral retina refraction is time-consuming, some previous studies used the summed RCRPS as an index to reflect myopic retinal defocus indirectly. In eyes with different SE across patients, the axial growth has been reported to be proportional to that of the RCRPS [29–31]. Therefore, we hypothesize that the interocular difference in RCRPS (dRCRPS) might be one of important factors involved in orthokeratology treatment that leads to changes in the magnitude of anisometropia. The present study aims to analyze the between-eye differential axial growth in anisometropes wearing orthokeratology lenses, and examine its association with dRCRPS. Furthermore, building upon our previous study where we found that baseline ocular characteristics, including axial length (AL) and SE at baseline, influence the efficacy of orthokeratology [32], we will analyze the extent to which between-eye difference in baseline AL and initial SE contribute to the interocular difference in ALG of anisometropes treated with orthokeratology.

## Methods

### Subjects

Subject recruitment was conducted at the Tianjin Medical University Eye Hospital between June 2017 and May 2018. The inclusion criteria were ages between 8 and 14 years; cycloplegic SE from –0.75D to –5.00D; initial anisometropia no less than 1.00 D; with-the-rule astigmatism of < 1.50 D; best-corrected monocular optical acuity of ≥ 20/20; myopia in both eyes and first time to get corrected with orthokeratology lens or spectacles, no strabismus or ocular surface disease; and no history of surgery or contact lens wearing.

A total of 102 subjects with anisometropia were included in this prospective interventional study. Sixty-one subjects were randomly assigned to the orthokeratology group to have both eyes fitted with orthokeratology lenses. Forty-one subjects were randomly assigned to the spectacle group to have the refractive errors of both eyes corrected with spectacle lenses. The randomization scheme for the study was performed using a commercial spreadsheet generator (Excel; Microsoft, Redmond, WA). All subjects and their legal guardians were informed of the potential risks associated with the study, and written consent forms were obtained. This study adhered to the tenets of the Declaration of Helsinki and was approved by the Institutional Ethics Committee Review Board of Tianjin Medical University Eye Hospital (Permit Number: 2017KY(L)-37).

The baseline information is shown in Table 1.

**Table 1:** Baseline demographics data of the subjects

	Binocular OK (*n*=61)	Spectacle (*n*=41)	*p* value
Sex (M/F)	29/32	20/21	0.90
Age (year)	11.81±1.24	11.33±1.29	0.09
More myopic eye			
SE (D)	−4.42±1.45	−4.32±0.95	0.38
AL (mm)	25.17±0.90	25.02±0.99	0.17
Less myopic eye			
SE (D)	−2.82±1.25	−2.62±0.98	0.35
AL (mm)	24.65±0.95	24.56±0.93	0.19
Interocular difference			
SE (D)	−1.59±0.67	−1.40±0.56	0.11
AL (mm)	0.47±0.32	0.46±0.36	0.14

*M*, Male; *F*, female; *SE*, spherical equivalent; *AL*, axial length

### Lens fitting and follow-up visits

The subjects in the orthokeratology group were fitted with spherical 4-zone orthokeratology lenses (Euclid Systems Corporation, Herndon, USA) in both eyes. The lens was composed of oprifocon A (Boston Equalens II) and had an oxygen permeability (DK) of 127 × 10^−11^cm^2^/s (mL O_2_/mL· mmHg). The total lens diameter ranged from 10.2 to 11.0 mm, which was determined by the horizontal visible iris diameter from the corneal topography. The lens fitting procedures strictly followed the guidelines provided by the manufacturer. In detail, the first trial alignment curve for the lens was based on the flat-K, corneal eccentricity and the horizontal iris diameter extracted from the corneal topography. Fitting quality was evaluated by fluorescence staining 30 min after the lens insertion. A satisfactory lens fitting was indicated by an optical zone covering the pupil, no apparent decentration of the lens, blink lens movement less than 1 mm, and a classic bullseye pattern with fluorescence staining [33]. Corneal topography was performed 40 min after the optimum trial lens insertion to observe the relatively centered plus-power ring in the corneal tangential difference map. The same experienced optometrist performed the fitting procedure and determined the final prescription (alignment curve, target power and diameter). Lenses were ordered with over-correction targeted at + 0.75 D [34]. The mean target power was −4.37±1.61 D. Usually after 2 weeks, the lenses dispensing was conducted, and the subjects were instructed on contact lens wearing and cleaning. The subjects were required to wear the lenses for at least 8 h per night and at least 6 days per week. Follow-up visits were scheduled at 1 day, 7 days, and 1 month, and at least once every 3 months thereafter. Lens prescription was modified only when the unaided monocular visual acuity was less than 20/30 or significant lens decentration was found. The subjects in the spectacle group were fitted with ordinary and commercially available glasses, and were required to wear spectacles all day long, except for sleeping. The prescription was modified based on their visual acuity, refractive changes, and interpupillary distance changes when appropriate, and follow-up visits were scheduled every three months after glasses placement.

### Measurement of refraction, axial length, and corneal topography

Cycloplegic refraction was performed with compound tropicamide eye drops (5 mg/mL, one drop every 5 min for four times) in all the subjects. Cycloplegic SE was calculated as the spherical power plus one-half of the cylindrical power. Noncontact optical biometry (Lenstar 900; Haag-Streit AG, Switzerland) was used to measure AL in all subjects at baseline and at 12-month visit after treatment. All inspections were performed by the same experienced technician, and the result met the quality control requirements of the instrument. At each visit, three consecutive measurements were collected, and the average was calculated and recorded.

**Fig. 1:**
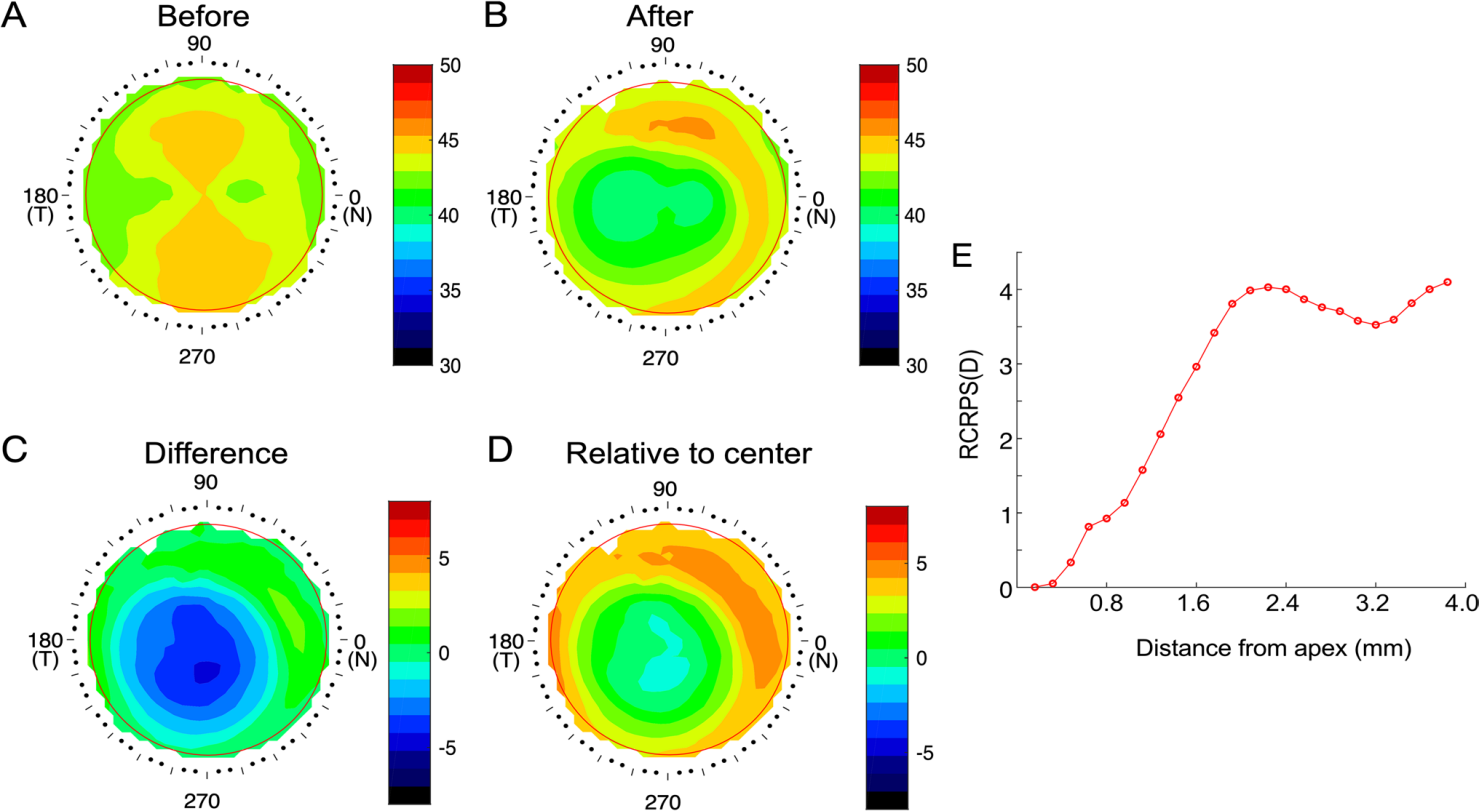
The RCRPS analysis. **A** Axial maps at baseline. **B** Axial maps 12 months after treatment. **C** CRPS derived by subtracting the baseline maps from the after-treatment maps. **D** RCRPS derived by normalizing the CRPS to the apex. **E** A representative RCRPS profile derived by taking the mean values of the points with the same radius

Corneal topography was obtained with Medmont (Medmont Pty Ltd. Camberwell, Victoria, Australia) at baseline and at every follow-up visit. At least four maps were measured, and best-quality maps were used for the analysis. Best-quality maps were defined those with a uniform and stable tear film during measurement, and a vertical width of the map ≥ 8 mm. The axial maps before treatment (baseline, Fig. 1A) and at the 12-month follow-up visit (after treatment Fig. 1B) were used in analysis with a custom MATLAB function. A corneal refractive power shift (CRPS) map (Fig. 1C) was first obtained by subtracting the baseline map from the after-treatment map. Then, the apex value was subtracted from each point of the CRPS map to derive a relative corneal refractive power shift (RCRPS) map (Fig. 1D). The RCRPS profile was derived by taking the mean values of the points with the same radius (Fig. 1E).

### Statistical analyses

For descriptive purposes, the means and standard deviations of all the parameters measured were calculated. The normality of the data was tested using the Shapiro–Wilk test. When normality was not rejected, comparisons between two groups were performed using A paired *t* test. Chi-square test was performed to compare the male/female ratio (M/F ratio) difference between two groups. Independent variables includes the interocular difference in baseline SE, the interocular difference in baseline AL, and the interocular difference in RCRPS (dRCRPS). Univariate linear regression showed the relationship between the interocular difference in ALG and each of the independent variables. Multivariate regression was used to analyze the contribution of the variables on the intraocular difference in ALG. All the statistical analyses were performed using the R software (https://www.r-project.org/). A *p* value of < 0.05 was considered as statistically significant.

## Results

### Interocular difference in AL

**Fig. 2:**
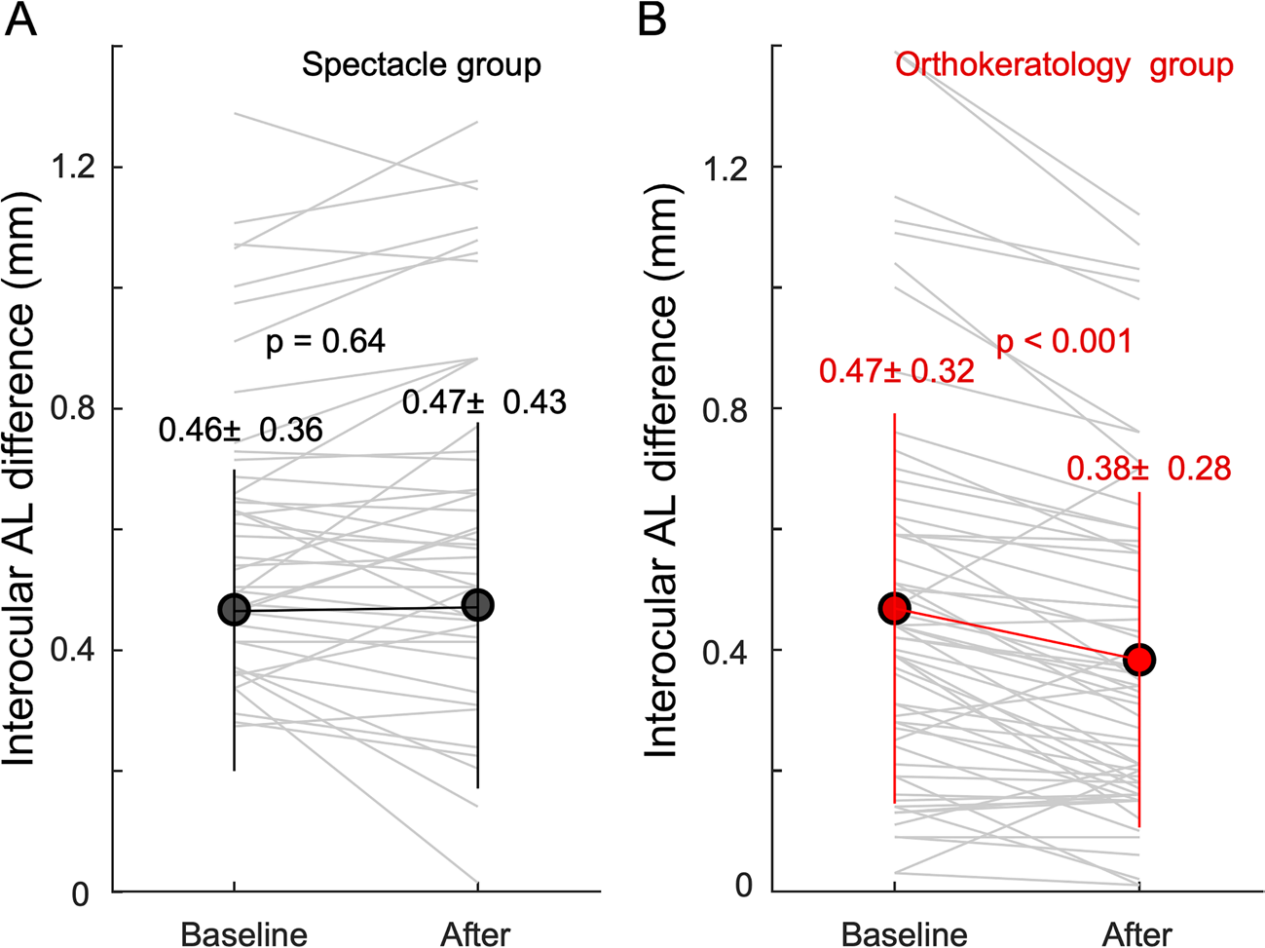
Interocular difference in AL at baseline and one year after treatment. **A** Spectacle group. **B** Orthokeratology group

In the control subjects wearing spectacles, the interocular difference in AL did not change significantly from baseline to post-treatment, 0.46 ± 0.36 vs. 0.47 ± 0.43 mm (*p* = 0.64, Fig. 2A). In the subjects treated with orthokeratology lenses, the interocular difference in AL significantly decreased 1 year after lens-placement (baseline vs. after treatment: 0.47 ± 0.32 vs. 0.38 ± 0.28, *p* < 0.001; Fig. 2B).

### Interocular difference in ALG

**Fig. 3:**
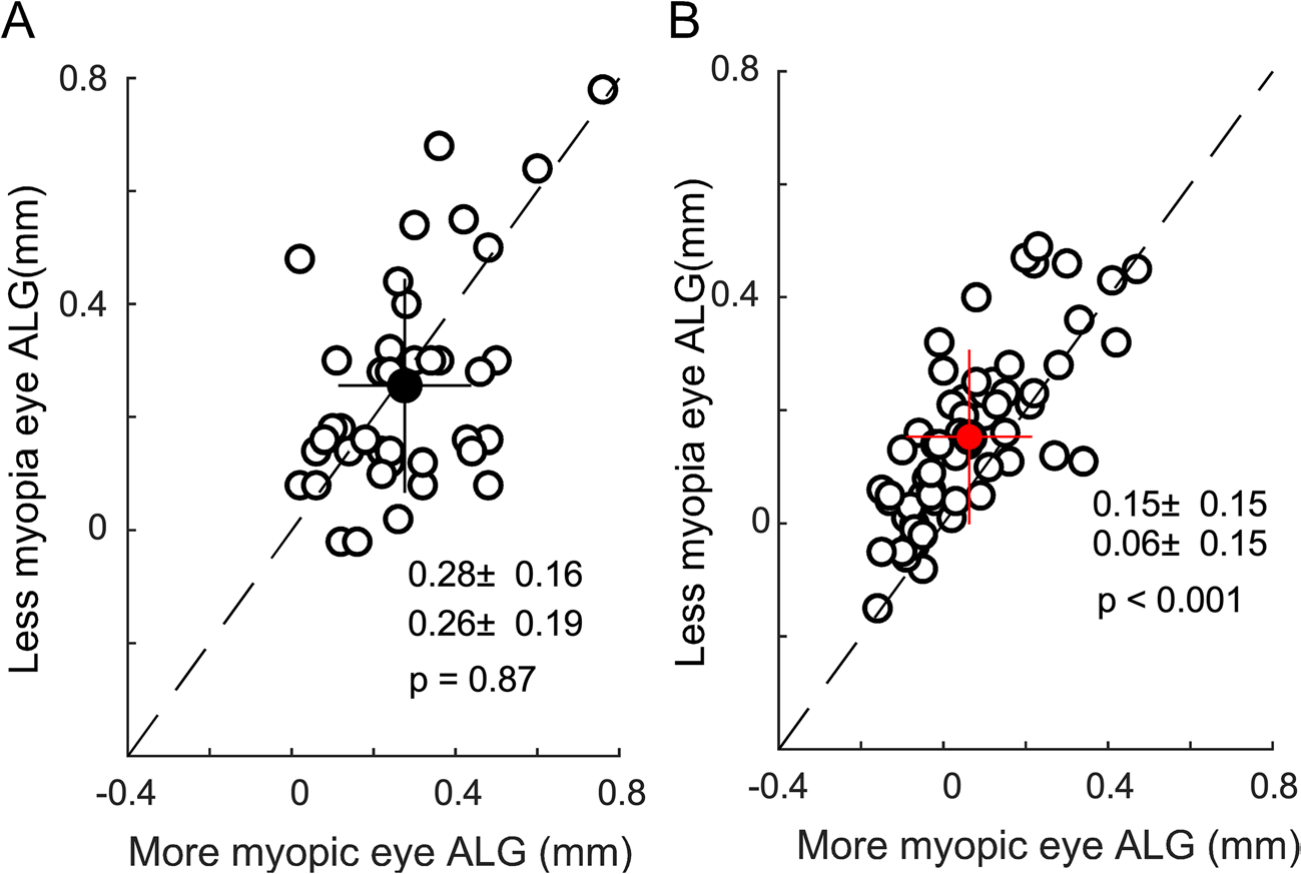
ALG in more and less myopic eyes in one-year follow-up. **A** Spectacle group. **B** Orthokeratology group

In the spectacle group, ALGs were similar in both eyes after one year (more vs. less myopic eyes: 0.26 ± 0.19 mm vs. 0.28 ± 0.16 mm, *p* = 0.87; Fig. 3A). In the orthokeratology group, the less myopic eyes had significantly larger ALG (0.15 ± 0.15 mm) than the more myopic eyes over 1-year follow-up (0.06 ± 0.15 mm, *p* < 0.001; Fig. 3B).

### Association between interocular difference in ALG and interocular difference in baseline SE

**Fig. 4:**
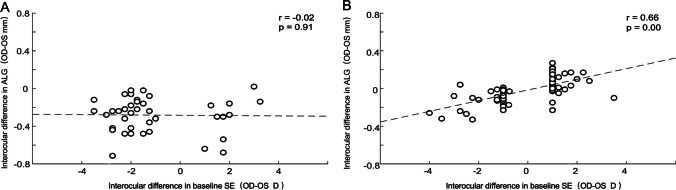
The association between interocular difference in ALG and interocular difference in baseline SE. **A** Spectacle group. **B** Orthokeratology group

Univariate linear regression revealed that the interocular difference in ALG in the spectacle group was not associated with the interocular difference in baseline SE (*r* = –0.02, *p* = 0.91; Fig. 4A). In the orthokeratology group, the interocular difference in ALG was significantly correlated with the interocular difference in baseline SE (*r* = 0.66, *p* < 0.001; Fig. 4B), indicating the greater the difference in degree of baseline myopia between two eyes undergoing orthokeratology, the greater the inter-eye difference in ALG.

### Association between interocular difference in ALG and interocular difference in baseline AL

**Fig. 5:**
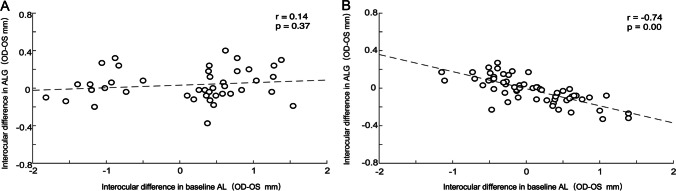
The association between interocular difference in ALG and interocular difference in baseline AL. **A** Spectacle group. **B** Orthokeratology group

Simple linear regression revealed a significant association between the interocular difference in ALG and the interocular difference in baseline AL in the orthokeratology group (*r* = −0.74, *p* < 0.001; Fig. 5B) but not in the spectacle group (*r* = 0.14, *p* = 0.37; Fig. 5A). Children in the orthokeratology group with a greater difference in baseline AL experienced a bigger interocular difference in ALG during the 1-year follow-up.

### RCRPS in the orthokeratology group

**Fig. 6:**
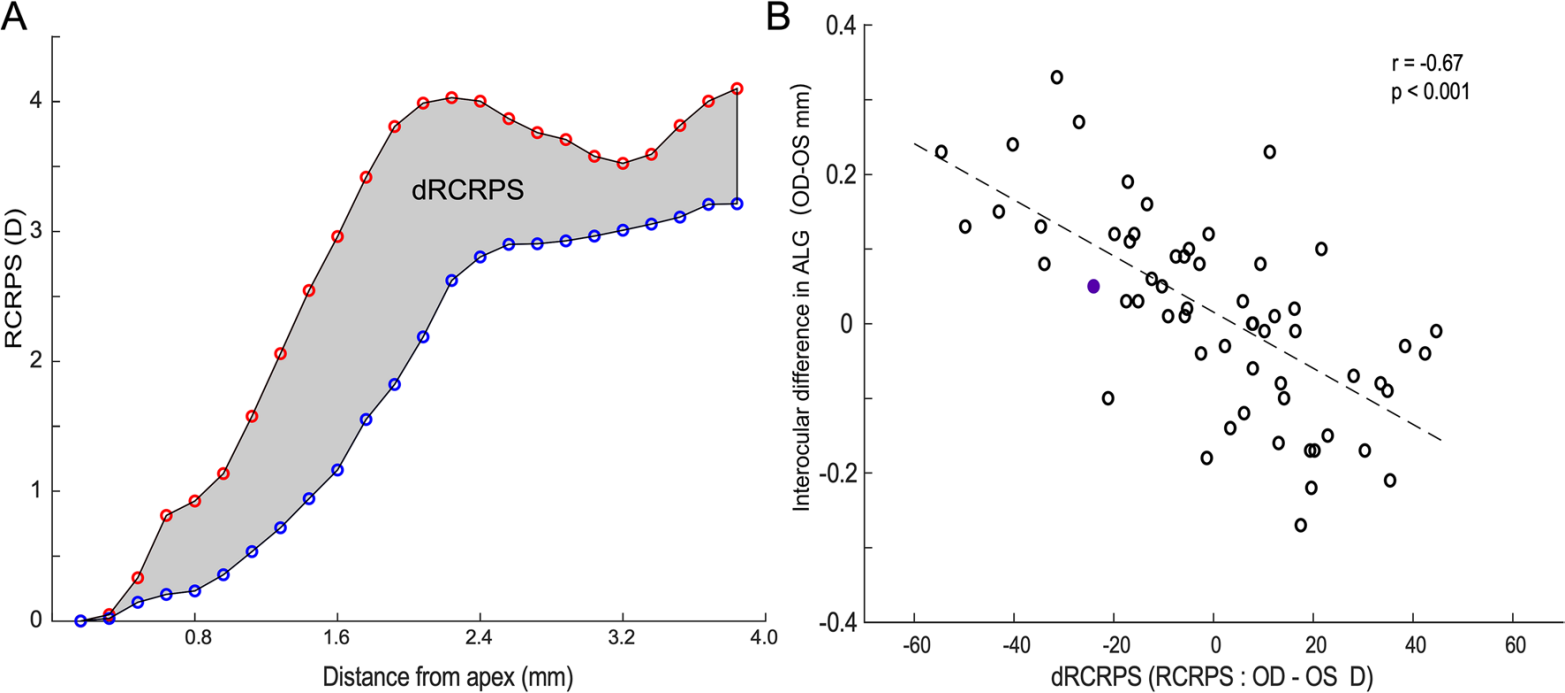
RCRPS and ALG. **A** RCRPS profiles of the right (red) and left eye (blue) in a subject treated with orthokeratology lenses. The shadowed area between the profiles represents the interocular difference in RCRPS (dRCRPS). **B** The association between the interocular difference in ALG and dRCRPS (shaded area in panel A)

The alteration of the corneal front surface led to a RCRPS, which was summed over a region with a diameter of 8 mm (Fig. 5A). A significant correlation was found between the interocular difference in RCRPS (dRCRPS) and the interocular difference in ALG at 1-year follow-up (*r* = –0.67, *p* < 0.001; Fig. 6B). dRCRPS captured 44.89% of the variance of the interocular difference in ALG (*p* < 0.001).

### Multiple regression analysis

Multiple regression analysis revealed that of the parameters included, the interocular difference in ALG was significantly associated with dRCRPS (*β* = −0.003, *p* < 0.001) and the interocular difference in baseline AL (*β* = −0.1179, *p* < 0.001), but not with the interocular difference in baseline SE (Table 2). The inclusion of the interocular difference in baseline AL captured more variance in the interocular difference of ALG (an increase to 61.97% from 44.89%).

**Table 2:** Multivariable regression analysis showing the association between interocular difference in ALG and two variables: the interocular difference in baseline AL and dRCRPS

Variables	Beta	*p* value	95% confidence interval
Interocular difference in baseline AL	−0.1179	< 0.001	−0.1715 to −0.0642
dRCRPS	−0.0030	< 0.001	−0.0032 to −0.0008
Model	*R*^2^ = 0.6197		

*AL*, Axial length; *ALG*, axial length growth; *dRCRPS*, the interocular difference in RCRPS

## Discussion

In children with myopic anisometropia treated with orthokeratology lenses, we found that the ALG in the relatively less myopic eyes exceeded that in the more severely myopic eyes. Notably, a distinct and statistically significant dose-response correlation was identified between the interocular discrepancy in ALG and the interocular difference in RCRPS (dRCRPS), indicating that eyes which underwent more relative corneal power change following orthokeratology treatment would achieve less axial growth. Based on these findings, it may be possible to enhance the ALG inhibition in the less myopic eyes of anisometropic children undergoing orthokeratology treatment by modifying the lenses to increasing RCRPS.

### Connections to previous studies

In this study, the anisomyopic spectacle-wearers exhibited relatively fast ALGs in both the more myopic eyes and the fellow less myopic eyes. The ALGs were comparable between two eyes, 0.28 mm for the more myopic eye and 0.26 mm for the less myopic eyes. These values were in line with those previously reported of approximately 0.27–0.37 mm per annum following wearing spectacles [9, 12, 26, 35–37]. In contrast, our study found significantly smaller ALGs in the orthokeratology group, with the more myopic eye exhibiting an increase of 0.06 mm/year and the less myopic eye exhibiting an increase of 0.15 mm/year. These findings were in accordance with those reported in previous studies showing ALGs of 0.03–0.09 mm/year and 0.1–0.15 mm/year in the more and the less myopic eyes, respectively, for bilateral myopic anisometropes [18, 22, 23]. Moreover, a remarkable reduction of 0.09 mm in the inter-eye AL difference from baseline to 12-month visit in subjects treated with orthokeratology lenses was found here, which was supported by previous reports of reductions ranging from 0.04 to 0.12 mm [18, 20, 21, 23, 24, 26, 38]. Nevertheless, Zhang et al. [39] reported no significant efficacy in controlling interocular differences in AL during binocular wearing of orthokeratology lenses, inconsistent with what was discovered in this manuscript. These disparate results may be attributable to several variables, such as the degree of anisometropia and the usage of different types of orthokeratology lenses. The magnitude of anisometropia is correlated with the severity of myopia in both eyes, and it typically increases with myopia progression [19, 40, 41]. Therefore, the likelihood of a spontaneous decrease in the degree of anisometropia with age is low [19, 41], as confirmed by the ALG values reported in the subjects who wore spectacles. The retardation of ALG and reduction in the magnitude of anisometropia can largely be explained as effects of the orthokeratology treatment [22].

### Relative corneal refractive power shift in myopic anisometropes underdoing orthokeratology

The orthokeratology lens shifted the peripheral defocus to myopic by inducing an RCRPS. Previous study has reported that RCRPS could capture approximately 10% of the variance observed in ALG [31], but this finding was based on ALGs in different isomyopic subjects, not in both eyes of subjects with anisometropia. For bilateral myopic anisometropes, here, we found that the RCRPS could capture around 7% of the variance observed in ALG whether in more myopic eyes or in less myopic eyes. Therefore, intersubject variables such as sex, age, genetic and environmental factors, and reading habits [9, 12, 35, 36, 42–44], pupil size [42, 45, 46], and corneal asymmetry [3, 45], might account for the low proportion of variance explained. To address this, we applied a within-person interocular comparison to explore the dose-response between RCRPS and ALG. After controlling for the intersubject variables, linear regression revealed that the interocular difference in RCRPS could capture approximately 44.89% of the variance of the interocular difference in ALG. Interocular difference in RCRPS represents the difference in the front surface of the eye. However, at the back side, eyes started with different baseline ALs, which affect the ALG in myopic children treated with orthokeratology lenses [44, 47]. Therefore, we added the interocular difference in baseline AL into the regression model, and the captured variance of interocular difference in ALG increased to 61.97%. The study by Xu et al. [38] corroborate this point by demonstrating that the initial inter-eye AL difference was associated with the change of AL difference between two eyes of myopic anisometropia patients who used binocular orthokeratology lenses, but without mention the role of the induced RCRPS. This study is the first to propose that the interocular variation in RCRPS had a great influence on the rates of AL progress in two eyes, and subsequently contributes to the reduction of anisomyopia in children post-orthokeratology. The findings of Zhong et al. ^22^ and Hu et al.^24^ appeared to support this view. They reported that the summed RCRPS achieved at early post-orthokeratology was negatively correlated with ALG. Moreover, it seemed that children with higher baseline SE were more liable to obtain greater areal summed RCRPS [31]. And higher baseline SE of children or target power of orthokeratology lenses was closely correlated with the lower increase of ALG [35, 48]. However, using multiple regression, we found that the contribution of the inter-eye difference in baseline SE on the interocular difference in ALG for myopic anisometropes wearing orthokeratology lenses was not statistically significant, indicating that there have been some situations illustrating substantially different initial myopia but comparable summed RCRPS. Additionally, after orthokeratology lens wear, treatment zone decentration is common and unavoidable [49, 50]. Chen et al. proposed that different magnitudes of treatment zone decentration caused diversified changes in corneal refractive power [51]. Previous study has found a positive correlation between the treatment zone decentration and the summed RCRPS after orthokeratology treatment [33]. In this sense, the interocular variation in RCRPS of anisometropic children may be partly derived from differences in orthokeratology lens parameters that were used for each eye.

### Residual variance and other interocular differences

Despite the improved sensitivity of between-eye comparison, still an approximately 38% variance remains unaccounted. Other interocular differences in the altered corneal surface may also contribute to the residual variance. In the light path, lens and accommodation also contribute to retinal defocus. Several previous studies have investigated the accommodative changes following orthokeratology treatment. Gifford et al. [52, 53] discovered that patients treated with orthokeratology had better accommodative responses and less accommodative lag than those treated with single-vision contact lenses. Han et al. [54] found that accommodative accuracy and facility were improved with long-term orthokeratology. Improving accommodation, particularly reducing accommodative lag (axial hyperopic blur) during near-work, has the potential to slow myopia progression [55–57]. Anisometropia is a binocular abnormality, which has close association with neural asymmetry, such as ocular dominance, which refers to a preference for the visual inputs from one eye over the other [58, 59]. Thus, we considered that ocular sensory dominance might be another factor contributing to the residual variance. Around 60% of non-anisometropic people have clear ocular dominance. This number increases to 80.7% in anisometropic persons. More importantly, the more myopic eye of anisometropes is usually the sensory dominant eye [60]. Foutch et al. has found that the dominant eye was more sensitive overall than the non-dominant eye to grating stimuli [61]. Therefore, we speculated that the more myopic eye as the dominant eye might respond better to the defocus cue induced by orthokeratology treatment than the less myopic eye, thus resulting in slower AL elongation, which might partly explain the reduction in the magnitude of anisometropia. However, in this study, we did not assess the subjects’ ocular dominance. It is unknown whether the ocular dominance changes or even shifts between the eyes, as the magnitude of anisometropia is reduced during the orthokeratology treatment.

### Limitations of the present study

In this study, the peripheral retinal defocus was not directly measured. Instead, RCRPS was utilized as an indirect representation of retinal defocus. However, the exact relationship between RCRPS and retinal defocus has yet to be elucidated. A future study with measured peripheral retina defocus would provide a more comprehensive understanding of whether the relationship between ALG and peripheral myopic defocus is dose-dependent [22]. Furthermore, the present study has a relatively shorter follow-up duration of only one year. In future studies, longer durations and more observation points should be considered. The inclusion of binocular vision measurements, may offer new insights into the understanding of the differential axial growth observed in children with anisometropia treated with orthokeratology lenses.

## Conclusion

Orthokeratology is an effective treatment for reducing the degree of anisometropia by exerting greater control over the more myopic eye. A clear dose-response relationship was observed between the interocular difference in RCRPS and the interocular difference of ALG in children with myopic anisometropia treated with bilateral orthokeratology. In fact, a major portion of the variance in the interocular difference in ALG could be explained by the combination of interocular differences in RCRPS and baseline AL. However, eye-specific modifications of orthokeratology lens-fitting for myopic anisometropia eyes require further exploration, particularly with regard to their efficacy for myopia control and binocular visual function.

## Data Availability

Data are available upon request.
